# CircRNAs as biomarkers of cancer: a meta-analysis

**DOI:** 10.1186/s12885-018-4213-0

**Published:** 2018-03-20

**Authors:** Miao Wang, Yuxi Yang, Jian Xu, Wen Bai, Xueli Ren, Huijian Wu

**Affiliations:** 1 School of Life Science and Biotechnology, Dalian University of Technology, No.2 Linggong Road, Dalian, Liaoning Province 116024 China; 2 Physical Education Department, Dalian University of Technology, No.2 Linggong Road, Dalian, Liaoning Province 116024 China

**Keywords:** CircRNA, Cancer, Biomarker

## Abstract

**Background:**

The expression of circular RNA (circRNA) may affect tumor progression. However, there have been no systemic meta-analysis for cancer diagnosis by using circRNAs in clinical till now. Herein, we aimed to collect and examined all the evidence on the potential role of circRNA as novel biomarker in human cancers.

**Methods:**

A comprehensive search strategy was used to search relevant literatures in the databases of PubMed, Embase, and the Web of Science from 2015 to August 2017. The correlation between circRNA expression and the diagnostic accuracy of tumor markers was analyzed. The methodological quality of each study was assessed by quality assessment for the diagnostic accuracies of the eligible studies (QUADAS-2). Statistical analysis was performed by applying the STATA (version 12.0) software.

**Results:**

The present meta-analysis included 1752 patients with circRNA expression data of tumor and paired adjacent non-tumorous tissues from 17 publications (19 studies). The pooled sensitivity, specificity, positive likelihood ratios (PLR), negative likelihood ratios (NLR), and diagnostic OR (DOR) with their 95% confidential intervals (95%CIs), and AUC values were 0.72 (0.67–0.76), 0.74 (0.69–0.78), 2.80 (2.40–3.10), 0.38 (0.33–0.44), 7.00 (6.00–9.00), and 0.79, respectively. Subgroup analyses showed that the expression of circRNA in tissues of hepatocellular carcinoma (HCC) group was more prone to be detected than other tumor types, with a high values of the specificity, DOR, and AUC.

**Conclusions:**

circRNAs might be suitable as diagnostic biomarkers for tumors, especially in HCC diagnosis. Further prospective studies on the diagnostic value of circRNAs from the different tumors are needed in the future.

**Electronic supplementary material:**

The online version of this article (10.1186/s12885-018-4213-0) contains supplementary material, which is available to authorized users.

## Background

Cancer is a major public health problem worldwide [1, 2]. Liver cancer is the most commonly diagnosed cancer and the leading cause of cancer-related death in China, followed by lung and stomach cancer [3]. Strengthening the methods for early diagnoses of cancer, as well as improved treatments, will be of great importance to reduce the death rate [4]. Although the diagnostic tool for early cancer detection could reduce the mortality, there have been still a lack of effective biomarkers that can be used for early diagnosis of cancer. Nowadays, the gold standard for cancer tissue detection is the histological evaluation of biopsy [5]. Histological grade was evaluated following the National Comprehensive Cancer Network clinical practice guideline of oncology (V.1.2011). Though it is the most reliable way in cancer prediction with relatively high sensitivity and specificity, its usage is still limited in clinical for the pain of patients.

There are many clinical biomarkers, such as CEA and CA199, for tumor diagnosis, whereas their sensitivities and specificities are low [6, 7]. Therefore, it is necessary for us to investigate novel effective biomarkers for cancer diagnosis.

Circular RNA (circRNA) is a class of non-coding RNA that was discovered in eukaryotes in 1979 [8]. CircRNAs are generated from the back splicing of exons, introns, or both to form exonic or intronic circRNAs [9, 10]. In human cells, circRNAs are usually composed of 1–5 exons, and the exon 2 is often the upstream “acceptor” exon [11]. A genome-wide analysis found that at least 83% of the total circRNAs are overlapping with protein-coding regions [12]. The copy number of circRNAs can be up to ten times greater than that of associated linear RNAs, suggesting that these circRNAs possess potential biological functions rather than accidental errors during splicing [13].

Previously, circRNAs were found and considered to have no biological function [14]. In the twenty-first century, with the development of RNA deep sequencing technology and bioinformatics analyses, the abundance and diversity of circRNAs were identified. Numerous of studies have confirmed the functions of circRNAs in tumor cell proliferation, migration and invasion, which may be drawn into multiple types of cancer, including colorectal cancer (CRC), hepatocellular carcinoma (HCC), breast cancer, gastric cancer (GC), and so on [15–19].

Although their biological functions remain largely unknown, recent studies show that circRNAs have three main functions in mammalian cells [11, 15, 20]. First, circRNAs can regulate transcription and RNA splicing. Second, circRNAs function as microRNA (miRNA) sponges. Third, they can be translated into protein driven by N^6^-methyladenosine modification [21]. Taking advantage of RNA sequencing (RNA-seq) technology, the expressions of circRNAs were found to be dysregulated in multiple types of cancer cell lines, tumor tissues, and even plasma samples from patients, which correlated with certain clinical outcomes, suggesting the potential roles of circRNAs in tumor progression. Considering their conserved sequences and stable structures, circRNAs may potentially serve as required novel biomarker for cancer [22, 23].

In this article, we performed a meta-analysis to summarize the overall accuracy of circRNA in different types of cancers. The sensitivity and specificity of cirRNAs were evaluated to assess the feasibility as biomarkers of cancer diagnosis.

## Methods

### Search strategy

Sources of studies included the PubMed, Embase, and Web of Science online databases for the circRNAs research published in English until August 3, 2017. The key words we used in the research were “cancer” or “tumor” or “tumour” or “carcinoma” or “adenocarcinoma” and (“circRNA” or “Circular RNA”). Two researchers (Wang and Yang) searched the titles, abstracts, and full-text articles to choose the right article. Other researchers (Xu, Bai, and Ren) were involved in data extraction with the two previous researchers. Any disagreement was solved by a third researcher (Wu). Then extract the data from the selected article and fill it in the table.

### Inclusion and exclusion criteria

This study used the following criteria in the article selection.

All inclusion articles fitted the following criteria:

I. circRNAs and cancers were analysed their association.
II. The total number of sample, sensitivity, specificity, and AUC were available and/or data on dysregulated circRNAs were available.
III. The experimental and control group were available.
IV. Researches on circRNA in tissue or specimens in various tumor patients using qRT-PCR were included.

Therefore, all exclusion articles fitted the following criteria:

I. The subject of study was not human.
II. The article was not in English.
III. Tumor was not mentioned in the abstract.
IV. There was no comparative study.
V. In the absence of a control group reaserch.
VI. The article studied on circRNA in cell lines or peripheral blood including serum and plasma.
VII. Review, summary of conference, editorial, commentary, and without complete data article.

### Data extraction

The data are extracted according to different research types.

I. In circRNA tissue research, we extracted data involving type and number of sample and the cut-off value for upregulated and downregulated circRNA expression, and list of it.
II. In these researches using circRNAs as biomarkers for different type of cancer by qRT-PCR, we collected the sensitivity and specificity of the expression of circRNAs, and other information involving type and number of sample, the cutoff value, endogenous reference, and AUC
III. The previous research on circRNAs was associated with clinicopathological data, such as gender, age, tumor size, tumor number, differentiation, TMN stage, lymphatic metastases, vascular invasion, microscopic vascular invasion, CEA, CA19–9, and PNI in cancer patients.

### Statistical analysis

The analytical software STATA (version 1.4) was used to analyze the diagnostic value. The sensitivity, specificity, DOR, and AUC of circRNA associated with cancer diagnosis in each study. For the accuracy of the sample, the sensitivity, specificity, AUC, and DOR and their 95% confidence intervals (CI) were plotted A two-sided *p* < 0.05 was considered statistically significant. We have used a fixed-effect model in the minimal heterogeneity (I^2^ < 50%) and used a random-effects model in the significant heterogeneity (I^2^ > 50%). The possible source of heterogeneity was performed using regression analysis. Subgroup analysis was performed according to tumor type using sensitivity, specificity, PLR, NLR, and DOR. The sensitivity and specificity were calculated as the area under the curve (AUC).

## Results

### Search and description of studies

**Fig. 1:**
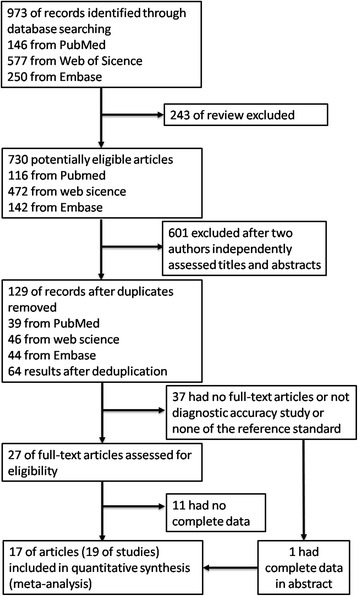
The flowchart shows the algorithm to identify inclusion articles. This study found 19 eligible articles that used circRNAs as biomarkers for tumor diagnosis by qRT-PCR

In the data, we analyzed the circRNA expression in various cancers in order to evaluate it as biomarkers of cancers. The characteristics of studies included in this analysis are summarized in Table 1 and Additional file 1: Table S1. One thousand seven hundred fifty-two tumor tissue specimens and paired adjacent non-tumorous tissues individuals from 19 studies published from 2015 to August 2017 were contained in this meta-analysis. The flow diagram for literature research processes was shown in Fig. 1.

**Table 1:** Characteristics of the included studies

	First author	Year	Ethnicity	Sample size		Cancer	Specimen	Method	Dignostic power				CircRNA
				Control	Case				*Sen*	*Spe*	AUC	Cutoff value	
1	Yao Z^1^	2017	Asian	102	102	hepatocellular carcinoma	tissue	qRT-PCR	0.822	0.724	0.834	–	circZKSCAN1
2	Fu L^2^	2017	Asian	102	102	hepatocellular carcinoma	tissue	qRT-PCR	0.716	0.815	0.848	–	hsa_circ_0004018
3	Qin M^3^	2016	Asian	89	89	hepatocellular carcinoma	tissue	qRT-PCR	0.81	0.69	0.63	0.0007855	hsa_circ_0001649
4	Shang X.C.^4^	2016	Asian	30	30	hepatocellular carcinoma	tissue	qRT-PCR	0.833	0.9	0.94	0.000586	hsa_circ_0005075
5	Fu L^5^	2017	Asian	107	107	hepatocellular carcinoma	tissue	qRT-PCR	0.449	0.868	0.7	12.24	hsa_circ_0003570
6	Shao YF^6^	2017	Asian	311	311	gastric cancer	tissue	qRT-PCR	0.646	0.698	0.72	9.125	hsa_circ_0000705
7	Lu R^7^	2017	Asian	96	96	gastric cancer	tissue	qRT-PCR	0.6	0.81	0.74	8.17	hsa_circ_0006633
8	Li P^8^	2015	Asian	101	101	gastric cancer	tissue	qRT-PCR	0.81	0.62	0.73	12.9	hsa_circ_002059
9	Li P^9^	2017	Asian	101	101	gastric cancer	tissue	qRT-PCR	0.88	0.56	0.82	12.9	hsa_circ_0000096
10	Li W.H.^10^	2017	Asian	76	76	gastric cancer	tissue	qRT-PCR	0.711	0.816	0.834	0.2269225	hsa_circ_00001649
11	Chen S^11^	2017	Asian	104	104	gastric cancer	tissue	qRT-PCR	0.721	0.683	0.75	6.83	hsa_circ_0000190
12	Tian M^12^	2017	Asian	108	108	gastric cancer	tissue	qRT-PCR	0.852	0.565	0.75	12.31	hsa_circ_0003159
13	Shao Y^13^	201,702	Asian	96	96	gastric cancer	tissue	qRT-PCR	0.5938	0.8125	0.696	12.14	hsa_circ_0014717
14	Shao Y^14^	201,704	Asian	96	96	gastric cancer	tissue	qRT-PCR	0.678	0.857	0.792	9.53	hsa_circ_0001895
15	Wang XN^15^	2015	Asian	31	31	colorectal cancer	tissue	qRT-PCR	0.68	0.73	0.788	6.04	hsa_circ_001988
16	Zhu X^16^	2017	Asian	49	49	lung adenocarcinoma	tissue	qRT-PCR	0.755	0.796	0.815	0.00101	hsa_circ_0013958
17	LuL1^17^	2017	Asian	51	51	breast cancer	tissue	qRT-PCR	0.65	0.69	0.71	14.84	hsa_circ_006054
18	Lu L2^17^	2017	Asian	51	51	breast cancer	tissue	qRT-PCR	0.69	0.71	0.78	8.95	hsa_circ_100219
19	Lu L3^17^	2017	Asian	51	51	breast cancer	tissue	qRT-PCR	0.63	0.63	0.64	14.24	hsa_circ_406697

*Sen*, sensitivity, *Spe* specificity, *AUC* area under the ROC curve

Among the 19 studies, 9 explore the association between circRNA expression and gastric cancer, 5 investigated that in hepatocellular carcinoma, and the other 5 focused on colorectal cancer (*n* = 1), lung adenocarcinoma (*n* = 1), and breast cancer (*n* = 3). In terms of samples, tissue from tumor tissue and paired adjacent non-tumorous tissue, and the number of each study range from 30 to 311 pairs. All studies used qRT-PCR detected circRNAs. Histopathological grading information was provided in all studies. We generalized circRNAs that were upregulated in 5 studies and downregulated in 14 of the 16 studies (Table 2).

**Table 2:** Expression of the most frequently altered circRNA in cancer detected by the qRT-PCR

Up-regulated circRNAs (*n* = 5)	Down-regulated circRNAs (*n* = 14)
hsa_circ_0005075, hsa_circ_0013958, hsa_circ_006054, hsa_circ_100219, hsa_circ_406697	circZKSCAN1, hsa_circ_0004018, hsa_circ_0005075, hsa_circ_001988, hsa_circ_002059, hsa_circ_0001649, hsa_circ_0000190, hsa_circ_0003159, hsa_circ_0014717, hsa_circ_0001895, hsa_circ_0000096, hsa_circ_0003570, hsa_circ_0000705, hsa_circ_0006633

All studies assessed the diagnostic value of circRNAs as biomarkers in tumor. Figure 1 showed search process briefly. The endogenous reference used in studies were not unified, GAPDH was used in all studies, except β-actin in one study [24].

The correlation between circRNAs expression and clinicopathological factors was shown in Table 3. The expression of circRNAs was not associated with age, gender, tumor size, tumor number, TMN stage, tumor differentiation, lymphatic metastasis, distal metastasis, microscopic vascular invasion (tumor invasion), CEA, CA19–9, and PNI. The circRNAs expression were also associated with their target genes or miRNA.

**Table 3:** Althered expression of circRNA associated with clinicopathological features from various tumor patients

Clinicopathological factors	Altered expression of circRNA	
	Up	Down
TNM stage	hsa_circ_0013958	circZKSCAN1, hsa_circ_0004018, hsa_circ_002059, hsa_circ_0000190, hsa_circ_0003159, hsa_circ_001471^a^, hsa_circ_0000096
Differentitation	–	hsa_circ_0004018, hsa_circ_001988, hsa_circ_0001895, hsa_circ_0003570 hsa_circ_00001649,
Tumor size (Diameter < 5 cm)	hsa_circ_0005075	hsa_circ_0004018, hsa_circ_0001649, hsa_circ_0000190, hsa_circ_0003570
Tumor number	–	circZKSCAN1
Distal metastasis	–	circZKSCAN1^b^, hsa_circ_002059, hsa_circ_0000190, hsa_circ_0003159, hsa_circ_0014717, hsa_circ_0000096, hsa_circ_0006633
Lymphatic metastasis	hsa_circ_0013958	hsa_circ_0000190,
MVI	–	circZKSCAN1, hsa_circ_0003570
Age	–	hsa_circ_002059
Gender	–	hsa_circ_002059, hsa_circ_0003159, hsa_circ_0000096
CEA	–	hsa_circ_0014717, hsa_circ_0001895, hsa_circ_0006633
CA19–9	–	hsa_circ_0000190, hsa_circ_0014717
PNI	–	hsa_circ_001988

*MVI* Microscopic vascular invasion *CEA* Carcinoembryonic antigen(tissue), *PNI* Perineural invasion, *CA19–9* Carbohydrate antigen 19–9

^a^Early and advanced grade, ^b^ Vascular invasion

In addition, all the studies independently scored the included studies based on QUADAS-2 (quality assessment of diagnostic accuracy studies-2) score system. Detailed results of the QUADAS-2 assessment are provided in the appendix. All studies showed risk of bias, because thresholds for index test positivity had been predefined, and all the patients had the same reference standard. Nineteen documents included in the gold standard and 19 are compared, but did not mention whether or not to use the blind method. All of them had relatively high quality in Additional file 2: Figure S1, indicating the relatively the reliable foundation of our analysis.

### Meta-analysis

**Fig. 2:**
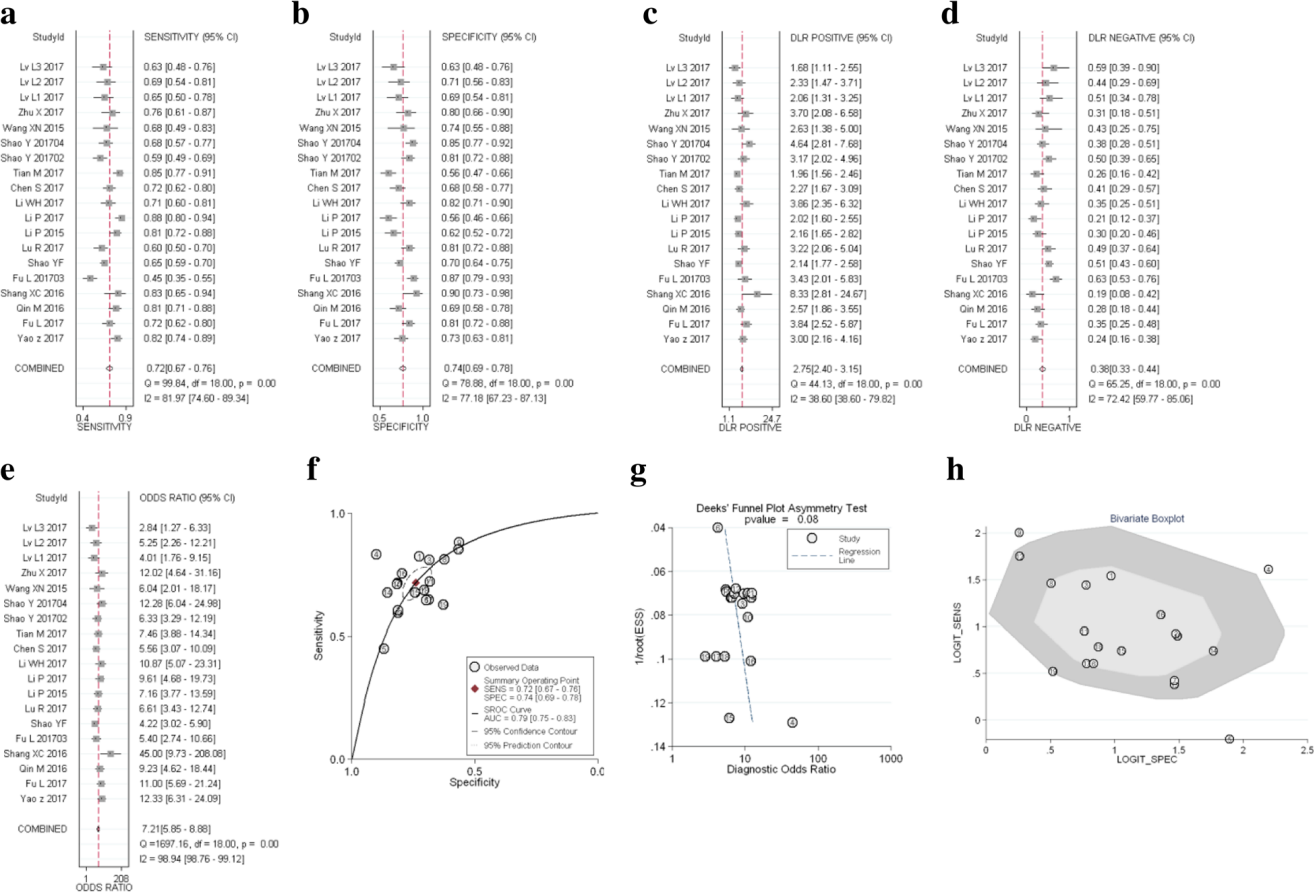
Forest plots of sensitivity, specificity, PLR, NLR, DOR, AUC, and funnel plot for diagnosis of circRNA in tumors among 19 studies. **a** Sensitivity; (**b**) Specificity; (**c**) PLR; (**d**) NLR; (**e**) DOR; (**f**) AUC; (**g**) Funnel plot; and (**h**) Bivariate boxplot

These 19 studies including 5 kinds of cancer and 1752 patients. A meta-analysis of the sensitivity, specificity, PLR, NLR, DOR, and summary receiver operator characteristics (SROC) for circRNAs was drew. Meta-analysis in significant heterogeneity (I^2^ > 50%) was taken by using a random effect model. The Q (Chi-square) value was 91.987 (*p* = 0.000) indicating substantial heterogeneity. The pooled sensitivity (Fig. 2a), specificity (Fig. 2b), PLR (Fig. 2c), NLR (Fig. 2d), and DOR (Fig. 2e) with their 95% confidential intervals (95%CIs), and value of AUC (Fig. 2f) for circRNA in 19 studies were 0.72 (95%CI:0.67–0.76), 0.74 (95%CI:0.69–0.78), 2.80 (95%CI:2.40–3.10), 0.38 (95%CI:0.33–0.44), 7.00 (95%CI:6.00–9.00), and 0.79 (95%CI:0.75–0.83), respectively. Furthermore, the funnel plot showed no publication bias (*p* = 0.083) (Fig. 2g). The results all together indicated a relatively moderate diagnostic accuracy of circRNA in detection of cancer patients.

### Meta-regression analyses

To find probable sources of heterogeneity, we used the meta-regression on the basis of types of cancer, endogenous reference and total sample size. Meta-regression analysis on the potential factors, which suggested that different types of sample were cause of heterogeneity (Table 4). A significant association for HCC (*p* = 0.0359) but not total sample size (*p* = 0.1876), and endogenous reference (*p* = 0.7341) were shown. And *p*-value of HCC groups was less than 0.05, which means the type of tumor is the reason for the formation of heterogeneity.

**Table 4:** Meta-regression circRNA associated with tumor type, sample size (> 50), and endogenous reference

Meta-Regression (Inverse Variance weights)					
Var	Coeff.	Std. Err.	*p*-value	RDOR	[95%CI]
Cte.	5.625	1.6550	0.0048	–	–
S	0.098	0.1198	0.4297	–	–
HCC	−0.810	0.3463	0.0359	0.44	(0.21;0.94)
GC	−0.402	0.2869	0.1844	0.67	(0.36;1.24)
size	−0.741	0.5329	0.1876	0.48	(0.15;1.51)
ref	−0.186	0.5345	0.7341	0.83	(0.26;2.64)

Tau-squared estimate = 0.0749 (convergence is achieved after 6 iterations)

Restricted Maximum Likelihood estimation (REML)

No. studies = 19

Filter OFF

Add 1/2 to all cells of the studies with zero

We also show the construction of a bivariate boxplot which is useful tool for the detection of heterogeneity for each study (Fig. 2h). There are 3 studies not located in the boxplot, including study 4, 5, and 9. Study 4 and 5 belong to HCC group, and study 9 in the GC group. That means tumor type is the major causes of heterogeneity.

We further excluded 1 study, which choose different endogenous reference (*p* = 0.7341), found by influence analysis and detection in Additional file 3: Figure S2. After exclusion, the sensitivity decreased from 0.72 to 0.71, the NLR increased from 0.38 to 0.39, and the specificity, DOR, PLR, and AUC not changed, showing minimal change with our overall analysis. We also further excluded 2 studies because of relatively small sample size (less than 50) (*p* = 0.1876), found by influence analysis and detection in Additional file 4: Figure S3. After exclusion, the sensitivity, PLR, DOR, and AUC not changed, specificity decreased from 0.74 to 0.73, the NLR increased from 0.38 to 0.39, and the specificity, DOR, PLR, and AUC not changed, showing minimal change with our overall analysis. The results confirmed that endogenous reference and sample size were not the cause for heterogeneity. We conducted subgroup analyses for the expression of circRNA in different type of tumor.

### Subgroup analysis

#### Hepatocellular carcinoma

**Fig. 3:**
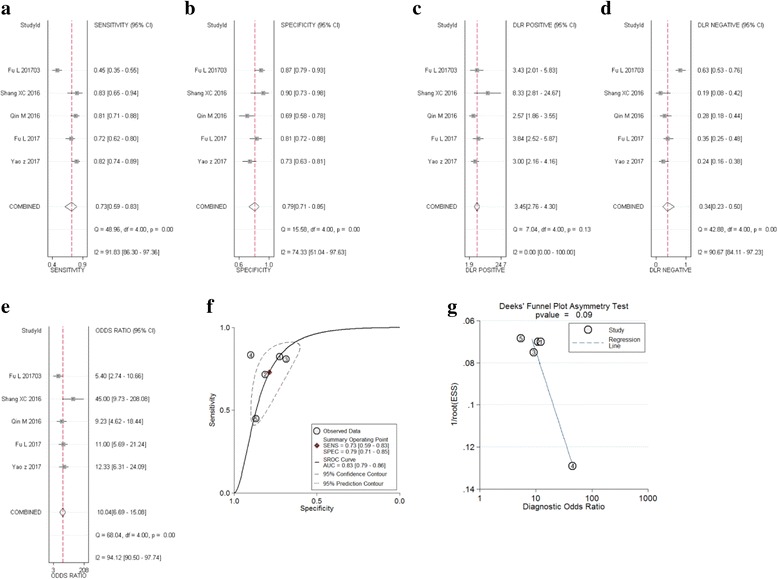
Forest plots of sensitivity, specificity, PLR, NLR, DOR, AUC, and funnel plot for diagnosis of circRNA in HCC among 5 studies. **a** Sensitivity; (**b**) Specificity; (**c**) PLR; (**d**) NLR. (**e**) DOR; (**f**) AUC; and (**g**) Funnel plot

Five studies including 430 patients analyzed the diagnostic value of circRNA in HCC. The meta-analysis of the sensitivity, specificity, DOR and AUC for circRNA in HCC. Meta-analysis was taken by using a random effect model (I^2^ > 50%). The pooled sensitivity (Fig. 3a), specificity (Fig. 3b), PLR (Fig. 3c), NLR (Fig. 3d), DOR (Fig. 3e), and AUC values (Fig. 3f) for circRNA levels in these studies were 0.73 (95%CI: 0.59–0.83), 0.79 (95%CI: 0.71–0.85), 3.40 (95%CI: 2.80–4.30), 0.34 (95%CI: 0.23–0.50), 10.00 (95%CI: 7.00–15.00), and 0.83 (95%CI:0.79–0.86), respectively. Furthermore, the funnel plot showed no publication bias (*p* = 0.092) (Fig. 3g).

#### Gastric cancer

**Fig. 4:**
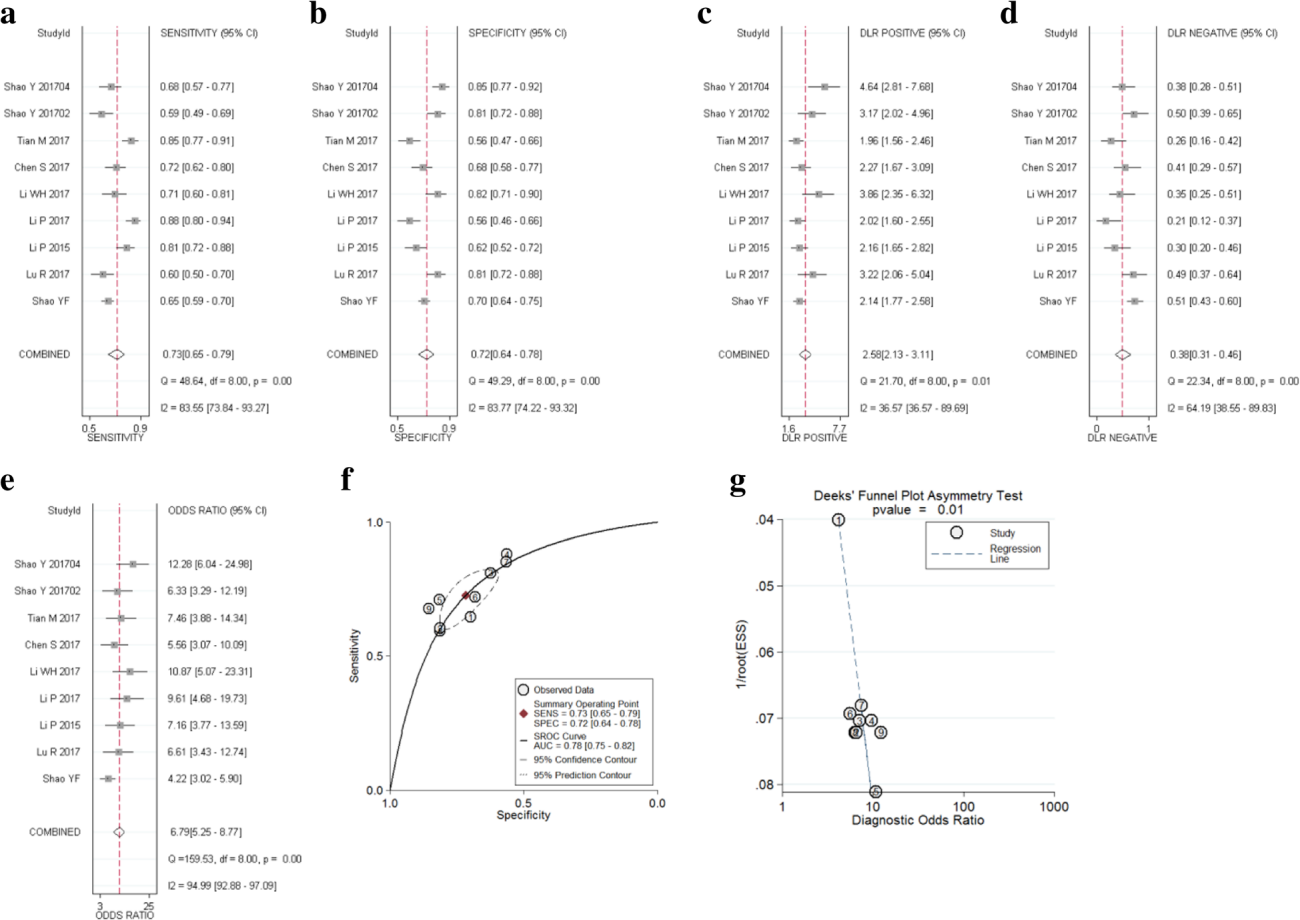
Forest plots of sensitivity, specificity, PLR, NLR, DOR, AUC, and funnel plot for diagnosis of circRNA in GC among 9 studies. **a** Sensitivity; (**b**) Specificity; (**c**) PLR; (**d**) NLR; (**e**) DOR; (**f**) AUC; and (**g**) Funnel plot

Nine studies including of 1089 patients investigated the diagnostic values of circRNA in gastric cancer (GC) and the meta-analysis of the sensitivity, specificity, DOR and AUC for circRNA in GC were plotted. Meta-analysis was taken by using a random effect model (I^2^ > 50%). The pooled sensitivity (Fig. 4a), specificity (Fig. 4b), PLR (Fig. 4c), NLR (Fig. 4d), DOR (Fig. 4e), *p*-values, and AUC values (Fig. 4f) for circRNA in these studies were 0.73 (95%CI: 0.65–0.79), 0.72 (95%CI:0.64–0.78), 2.60 (95%CI: 2.10–3.10), 0.38 (95%CI: 0.31–0.46), 7.00 (95%CI: 5.00–9.00), and 0.78 (95%CI:0.75–0.82), respectively. Furthermore, the funnel plot showed obviously publication bias (*p* = 0.006) (Fig. 4g).

#### Other tumors

**Fig. 5:**
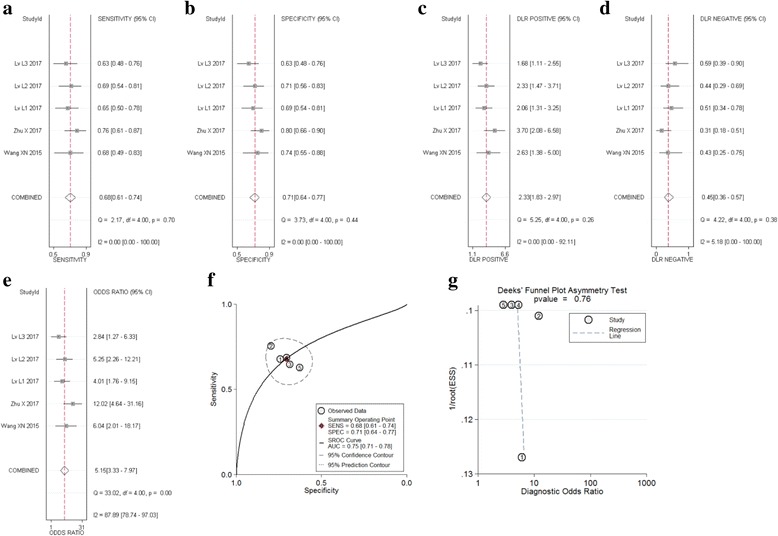
Forest plots of sensitivity, specificity, PLR, NLR, DOR, AUC, and funnel plot for diagnosis of circRNA in other type of tumors among 5 studies. **a** Sensitivity; (**b**) Specificity; (**c**) PLR; (**d**) NLR; (**e**) DOR; (**f**) AUC; and (**g**) Funnel plot

Five studies including 233 patients analyzed the diagnostic value of circRNA in colorectal cancer, lung adenocarcinoma, and breast cancer. The meta-analysis of the sensitivity, specificity, DOR and AUC for circRNA in these types of tumor. Meta-analysis was taken by using a random effect model. The pooled sensitivity (Fig. 5a), specificity (Fig. 5b), PLR (Fig. 5c), NLR (Fig. 5d), DOR (Fig. 5e), p-values, and AUC values (Fig. 5f) for circRNA in these studies were 0.68 (95%CI: 0.61–0.74), 0.71 (95%CI: 0.64–0.77), 2.30 (95%CI: 1.80–3.00), 0.45 (95%CI: 0.36–0.57), 5.00 (95%CI: 3.00–8.00), and 0.75 (95%CI: 0.71–0.78), respectively. All p-values (*p* = 0.495) more than 0.05 show that there’s no significant difference. But the sensitivity (0.68), specificity (0.71), and AUC (0.68) values than HCC and GC groups. And I^2^ = 0 show that there’s no significant heterogeneity. There is no difference between the fixed effect model and the random effect model. Furthermore, the funnel plot showed no publication bias (*p* = 0.762) (Fig. 5g).

## Discussion

In recent years, the detection of circRNAs expression in tumors was gradually recognized. However, there has been no meta-analysis on the expression of circRNAs in tumors. We collected published studies on the expression of circRNAs in various tumors, including 5 up-regulated circRNAs and 14 down-regulated circRNAs. There were four circRNAs including circZKSCAN1, hsa_circ_0004018, hsa_circ_0001649, and hsa_circ_0003570 down regulated and one circRNA (hsa_circ_0005075) up regulated in HCC, and nine circRNAs including hsa_circ_0000705, hsa_circ_0006633, hsa_circ_002059, hsa_circ_0000096, hsa_circ_00001649, hsa_circ_0000190, hsa_circ_0003159, hsa_circ_0014717, and hsa_circ_0001895 down regulated in gastric cancer, whereas three circRNAs including hsa_circ_006054, hsa_circ_100219, and hsa_circ_406697 were up regulated in breast cancer. Furthermore, circRNA hsa_circ_001988 down regulated in colorectal cancer and circRNA hsa_circ_0013958 up regulated in lung adenocarcinoma.

Only 5 studies noted that the patient accepted no adjunctive treatments before the surgery, involving chemotherapy, radiotherapy, and targeted therapy [25–28], and other studies not mentioned. We excluded some studies which were based on the expression of circRNA in cell lines, peripheral blood, serum, or plasma [29].

The diagnostic value of circRNA as a biomarker for cancer was assessed according to published studies. Sensitivity and specificity are statistical measures of the value of diagnostic. The DOR and value of AUC were used to describe the characteristics of index test and its suitability as a diagnostic method. As for the overall circRNAs expressions from all cancers, the sensitivity, specificity, PLR, NLR, DOR with the corresponding 95%CIs and AUC values were 0.72 (95%CI:0.67–0.76), 0.74 (95%CI:0.69–0.78), 2.8 (95%CI:2.40–3.10), 0.38 (95%CI: 0.33–0.44), 7.00 (95%CI: 6.00–9.00), and 0.79, respectively. The results showed above indicated that circRNAs are suitable for use as diagnostic biomarkers for cancer. However, it should be noted that there was substantial heterogeneity in the pooled estimates. We performed the meta-regression based on the variables including cancer type, endogenous reference, and total sample sizes. The result showed that the heterogeneity may come from the tumor types, but not the endogenous reference and sample sizes.

Three groups of tumors had been evaluated repeatedly, which were divided into three subgroups: HCC, GC, and other tumors group. In HCC group, the expression of circRNAs showed satisfactory values of sensitivity and specificity (sensitivity: 0.73; specificity: 0.79), and its specificity values was higher than the sensitivity values. The specificity value (specificity: 0.72) of GC group was lower than HCC group. In other types of tumors group, the value of sensitivity and specificity (sensitivity: 0.68; specificity: 0.71) were disappointing, because of the low sensitivity. It is possible that the circRNAs might be suitable for use as diagnostic biomarkers of cancer. The value of sensitivity, specificity, and AUC of GC group were higher than other types of tumors group. Among all circRNAs investigated in this study, circRNA hsa_circ_0005075 showed effective diagnostic accuracy (AUC: 0.94; sensitivity: 0.833; specificity: 0.90) in HCC group, and circRNA hsa_circ_0000096 showed effective diagnostic accuracy (sensitivity: 0.88; specificity: 0.56; AUC: 0.82) in GC group. Other types of tumors group may also be available, but more researches might be needed to prove it.

CircRNAs are abundant in the brain and exosomes, with the exception of tumors. Their capability to transverse the blood–brain barrier makes them perfect candidates as potential diagnostic tools for central nervous system (CNS) disorders [30]. A circRNA discovered in human named Cdr1as (antisense to the cerebellar degeneration-related protein 1 transcript), which also termed as ciRS-7 (circular RNA sponge for miR-7) aberrantly expressed in several cancers, including colorectal cancer [31], hepatocellular carcinoma [32], and gastric cancer [20], but these study did not have completed data for a meta analysis.

circRNAs have multiple biological functions [33]. (1) Transcription regulation. (2) Alternative splicing. (3) Regulation of parental gene translation. (4) Cell cycle regulation. (5) Protein sponge. (6) m6A-driven translation. (7) miRNA sponge. These suggested that the potential role of circRNAs in tumor progression. Detection of altered expression of circRNAs could have several advantages. First, the expressions of circRNAs were found to be dysregulated in multiple types of cancer cell lines, tumor tissues, and even plasma samples from patients. Second, circRNAs conserved sequences and stable structures. So circRNAs were deemed to be promising biomarkers for early diagnosis of cancer.

More importantly, the cancer profile in China is markedly different from those of developed countries. The diagnosis of the stomach and liver cancers in China comprise between one-third and one-half of the global incidence burden. The most common diagnostic in the United States is concentrated in prostate and breast cancers, however the expression of circRNAs in HCC and GC groups showed high values of sensitivity, specificity, and AUC. It indicates that the accurate diagnosis for the stomach and liver cancers is great social significance in China.

There are some deficiencies in the research. First, the samples are often lack of control of normal patients. Second, there is no complete data in some studies. We asked the author for data, but we did not get the detail data. Third, because the purposes of the study were different, some researches on the expression of circRNAs in cancer were excluded. We excluded some researches to ensure the accuracy of the analysis [32, 34–37]. However, more study is needed.

Although technology for RNA detection have became mature, the detection reagents tend to be expensive. There are some promising methods were used in miRNA detection, including bioluminescence and high-throughput sequencing [37, 38]. These detection technologies also can be used in circRNA detection, which might be improved before using in hospital. A quickly, accurately and cheaply detection method for circRNA may be used in hospitals in the future.

## Conclusion

It is possible that the circRNA may be suitable as a diagnostic biomarker for cancers, particularly in HCC tissues. The expression of circRNA detected may be applicable in gastric carcinoma, colorectal cancer, lung adenocarcinoma, and breast cancer, but more study is required to prove it.

## Additional files

Additional file1. **Table S1** All characteristics of all studies on the use of circRNAs as diagnostic biomarkers of cancer. (DOCX 100 kb)

Additional file 2. **Figure S1.** QUADAS studies of diagnostic test accuracy. (TIFF 1823 kb)

Additional file 3. **Figure S2.** Forest plots of sensitivity, specificity, PLR, NLR, DOR, AUC, and funnel plot for diagnosis of circRNA in tumors among 18 studies, which excluded different endogenous reference study. (A) Sensitivity; (B) Specificity; (C) PLR; (D) NLR; (E) DOR; (F) AUC; and (G) Funnel plot. (TIFF 1925 kb)

Additional file 4. **Figure S3.** Forest plots of PLR, NLR, DOR, AUC, and funnel plot for diagnosis of circRNA in tumors among 17 studies, which excluded 2 studies because of relatively small sample size (less than 50). (A) Sensitivity; (B) Specificity; (C) PLR; (D) NLR; (E) DOR; (F) AUC; and (G) Funnel plot. (TIFF 1858 kb)

## Abbreviations

AUC: Area under the Curve of ROC
CA19–9: Carbohydrate antigen 19–9
CEA: Carcinoembryonic antigen (tissue)
circRNA: Circular RNA
Cis: Confidential intervals
DOR: Diagnostic OR
GC: Gastric cancer
HCC: Hepatocellular carcinoma
MVI: Microscopic vascular invasion
NLR: Negative likelihood ratios
OR: Odds ratio
PLR: Positive likelihood ratios
PNI: Perineural invasion
qRT-PCR: Quantitative reverse transcription polymerase chain reaction
*sen*: Sensitivity
*spe*: Specificity
SROC: Summary receiver operator characteristics
