# Supplementary material for: CircRNAs as biomarkers of cancer: a meta-analysis
Source: BMC Cancer. 2018 Mar 20;18:303. doi: 10.1186/s12885-018-4213-0 (PMC5859638; doi:10.1186/s12885-018-4213-0)
Supplement: Supplementary file 1 — Table S1 All characteristics of all studies on the use of circRNAs as diagnostic biomarkers of cancer. (DOCX 100 kb) [file 12885_2018_4213_MOESM1_ESM.docx]

Supplementary table 1 All characteristics of all studies on the use of circRNAs as diagnostic biomarkers of cancer.

| **Author** | **Year** | **circRNA name** | **regulation** | **origin** | **Tumor size** | **Control size** | **Method** | **Endogenous reference** | **Specimen type and source** | **Nontumorous tissues selection** | **SEN** | **SPE** | **AUC** | **Cutoff** | **P value** |
| --- | --- | --- | --- | --- | --- | --- | --- | --- | --- | --- | --- | --- | --- | --- | --- |
| **Yao Z** | **2017** | **circ*ZKSCAN1*** | **down** | **The Second and The Third Affiliated Hospital of Sun Yat-sen University, China** | **102** | **102** | **qRT-PCR** | **GAPDH** | **hepatocellular carcinoma tissue specimens and paired adjacent non-tumorous tissues** | **5 cm from the edge of the cancer** | **0.822** | **0.724** | **0.834** | **-** | ***P*<0.05** |
| **Fu L** | **2017** | **hsa_circ_0004018** | **down** | **Ningbo No. 2 Hospital, Ningbo Lihuili Hospital and Ningbo Yinzhou Peoples’ Hospital, China** | **102** | **102** | **qRT-PCR** | **GAPDH** | **HCC and para-tumorous tissues** | **1 cm away from the edge of the HCC** | **0.716** | **0.815** | **0.848** | **-** | ***P*<0.001** |
| **Qin M** | **2016** | **hsa_circ_0001649** | **down** | **Zhongshan Hospital (Shang- hai, China)** | **89** | **89** | **qRT-PCR** | **β-actin** | **HCC and paired adjacent liver tis- sues** | **-** | **0.81** | **0.69** | **0.63** | **0.00** | **p=0.0014** |
| **Shang X** | **2016** | **hsa_circ_0005075** | **up** | **Shan Dong University affiliated with Shan Dong Provincial Hospital** | **30** | **30** | **qRT-PCR** | **GAPDH** | **HCC tissues and paired adjacent nontumorous tissues** | **-** | **0.833** | **0.9** | **0.94** | **0.00** | **P <0.001** |
| **Fu L** | **201703** | **hsa_circ_0003570** | **down** | **Ningbo No. 2 Hospital, Ningbo Li Hui-Li Hospital and Ningbo Yinzhou Peoples’ Hospital, China** | **107** | **107** | **qRT-PCR** | **GAPDH** | **HCC tissues and paired adjacent nontumorous tissues** | **1 cm away from the edge of the HCC** | **0.449** | **0.868** | **0.7** | **12.24** |  |
| **Shao YF** | **2017** | **hsa_circ_0000705** | **down** | **-** | **311** | **311** | **qRT-PCR** | **GAPDH** | **gastric cancer tissues and paired adjacent nontumorous tissues** | **-** | **0.646** | **0.698** | **0.72** | **9.125** |  |
| **Lu R** | **2017** | **hsa_circ_0006633** | **down** | **Affiliated Hospital of Ningbo University School of Medicine, China,** | **96** | **96** | **qRT-PCR** | **GAPDH** | **gastric cancer tissues and paired adjacent nontumorous tissues** | **5 cm from the edge of the tumor** | **0.6** | **0.81** | **0.74** | **8.17** | **P < 0.001** |
| **Li P** | **2015** | **Hsa_circ_002059,** | **down** | **Yinzhou People's Hos- pital and the Affiliated Hospital of Ningbo University, China** | **101** | **101** | **qRT-PCR** | **GAPDH** | **gastric cancer tissues and paired adjacent nontumorous tissues** | **5 cm from the edge of the cancer** | **0.81** | **0.62** | **0.73** | **12.9** | **P <0.001** |
| **Li P** | **2017** | **Circular RNA 0000096** | **down** | **The Affiliated Hospital of Ningbo University School of Medicine, China** | **101** | **101** | **qRT-PCR** | **GAPDH** | **gastric cancer tissues and paired adjacent nontumorous tissues** | **4 cm from the edge of the cancer** | **0.88** | **0.56** | **0.82** | **12.9** |  |
| **Li W.H.** | **2017** | **hsa_circ_00001649** | **down** | **First Affiliated Hospital of Xi’an Jiaotong University** | **76** | **76** | **qRT-PCR** | **GAPDH** | **Gastric tumor tissue samples and their paired paracancerous** | **-** | **0.711** | **0.816** | **0.834** | **0.2269225** | **P <0.01** |
| **Chen S** | **2017** | **hsa_circ_0000190** | **down** | **Ningbo Yinzhou People's Hospital, China** | **104** | **104** | **qRT-PCR** | **GAPDH** | **gastric cancer tissues and adjacent normal tissues** | **5 cm away from the edge of the gastric cancer** | **0.721** | **0.683** | **0.75** | **6.83** | **P <0.001** |
| **Tian M** | **2017** | **hsa_circ_0003159** | **low** | **The Affiliated Hospital of Ningbo University School of Medicine and Yinzhou People’s Hospital, China.** | **108** | **108** | **qRT-PCR** | **GAPDH** | **fresh gastric cancer tissues and adja- cent non-tumorous tissues** | **5 cm away from the cancer edge.** | **0.852** | **0.565** | **0.75** | **12.31** | **P <0.001** |
| **Shao Y** | **201702** | **hsa_circ_0014717** | **down** | **The Affiliated Hospital of Medical School of Ningbo University (China)** | **96** | **96** | **qRT-PCR** | **GAPDH** | **Gastric cancer tissues and their matched adjacent nontumorous tissues** | **5 cm away from the edge of tumor** | **0.5938** | **0.8125** | **0.696** | **12.14** | **P < 0.001** |
| **Shao Y** | **201704** | **hsa_circ_0001895** | **down-** | **The Affiliated Hospital of Ningbo University School of Medicine, China** | **96** | **96** | **qRT-PCR** | **GAPDH** | **gastric cancer tissues and their adjacent non-tumorous tissues** | **5 cm from the edge of tumor** | **0.678** | **0.857** | **0.792** | **9.53** | **p<0.001** |
| **Wang XN** | **2015** | **hsa_circ_001988** | **down** | **-** | **31** | **31** | ***real-time PCR*** | **GAPDH** | **colorectal cancer and adjacent normal mucosa)** | **-** | **0.68** | **0.73** | **0.788** | **6.04** | **p<0.05** |
| **Zhu X** | **2017** | **hsa_circ_0013958** | **up** | **Zhongda Hospital, china** | **49** | **49** | **qRT-PCR** | **GAPDH** | **lung adenocarcinoma** | **-** | **0.755** | **0.796** | **0.815** | **0.00101** | **P < 0.001** |
| **Lv L1** | **2017** | **hsa_ circ_006054** | **up** | **The Affiliated Hospital of Jiangsu University, the People's Hospital of Yixing and the First Affiliated Hospital of Suzhou University** | **51** | **51** | **qRT-PCR** | **GAPDH** | **Breast cancer lesions and adjacent normal- appearing tissues** | **>5 cm from the edge of the tumors.** | **0.65** | **0.69** | **0.71** | **14.84** | **P < 0.001** |
| **Lv L2** | **2017** | **hsa_ circ_100219** | **up** | **The Affiliated Hospital of Jiangsu University, the People's Hospital of Yixing and the First Affiliated Hospital of Suzhou University** | **51** | **51** | **qRT-PCR** | **GAPDH** | **Breast cancer lesions and adjacent normal- appearing tissues** | **>5 cm from the edge of the tumors.** | **0.69** | **0.71** | **0.78** | **8.95** | **P < 0.001** |
| **Lv L3** | **2017** | **hsa_ circ_406697** | **up** | **The Affiliated Hospital of Jiangsu University, the People's Hospital of Yixing and the First Affiliated Hospital of Suzhou University** | **51** | **51** | **qRT-PCR** | **GAPDH** | **Breast cancer lesions and adjacent normal- appearing tissues** | **>5 cm from the edge of the tumors.** | **0.63** | **0.63** | **0.64** | **14.24** | **p=0.008** |
