# Supplementary figures and images for: CircRNAs as biomarkers of cancer: a meta-analysis
Source: BMC Cancer. 2018 Mar 20;18:303. doi: 10.1186/s12885-018-4213-0 (PMC5859638; doi:10.1186/s12885-018-4213-0)

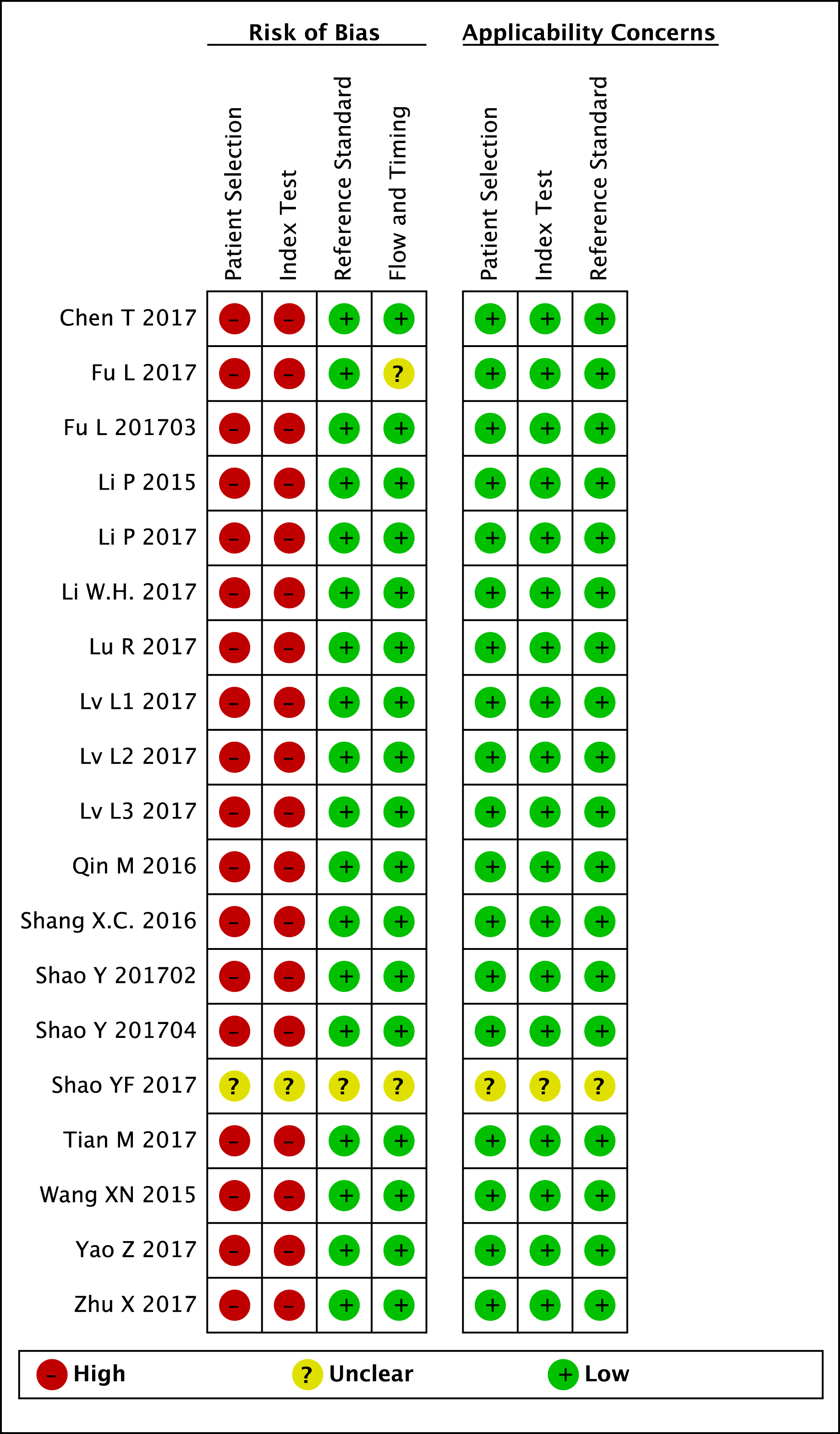

Supplement: Supplementary file 2 — Figure S1. QUADAS studies of diagnostic test accuracy. (TIFF 1823 kb) [file 12885_2018_4213_MOESM2_ESM.tif]

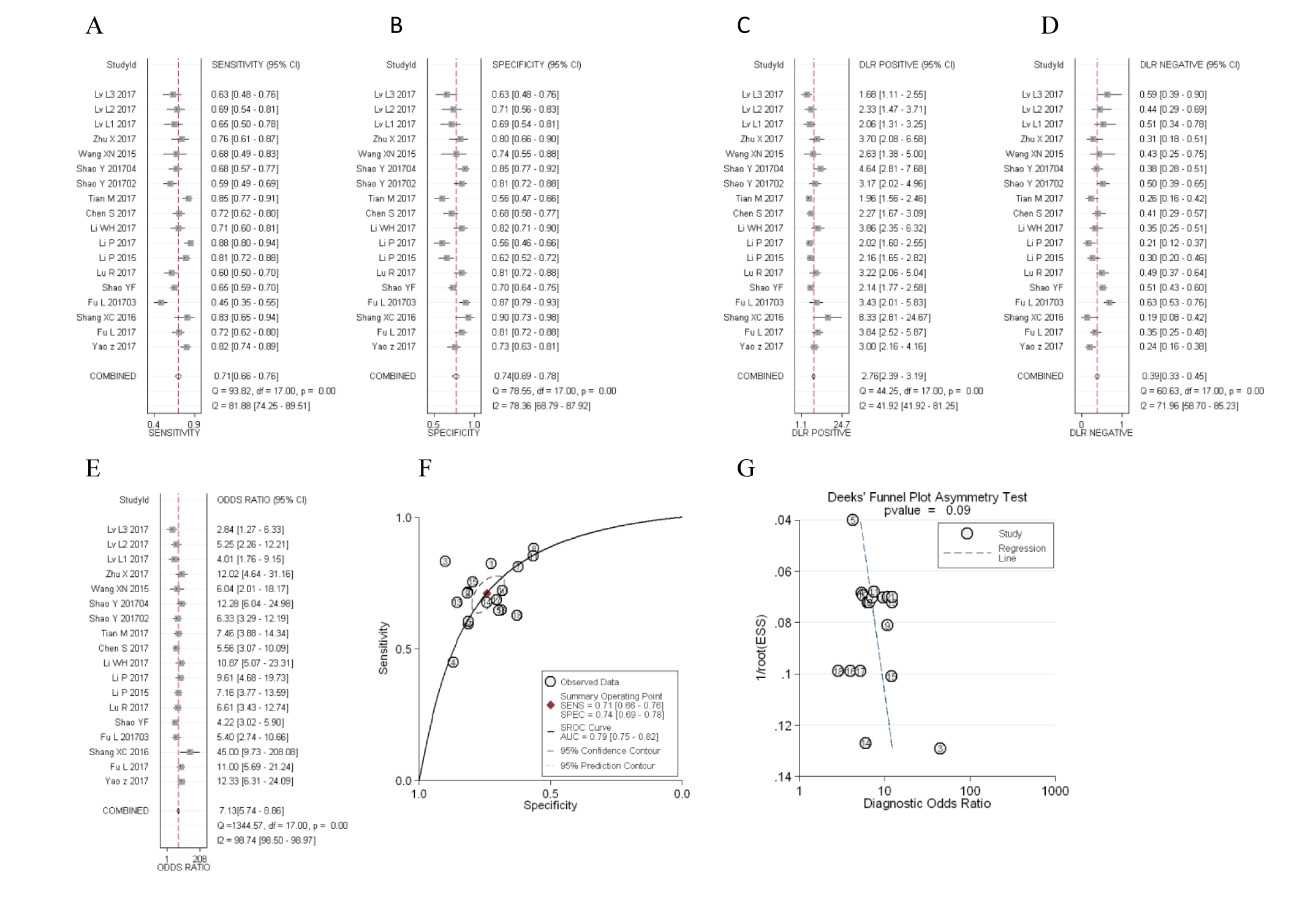

Supplement: Supplementary file 3 — Figure S2. Forest plots of sensitivity, specificity, PLR, NLR, DOR, AUC, and funnel plot for diagnosis of circRNA in tumors among 18 studies, which excluded different endogenous reference study. (A) Sensitivity; (B) Specificity; (C) PLR; (D) NLR; (E) DOR; (F) AUC; and (G) Funnel plot. (TIFF 1925 kb) [file 12885_2018_4213_MOESM3_ESM.tif]

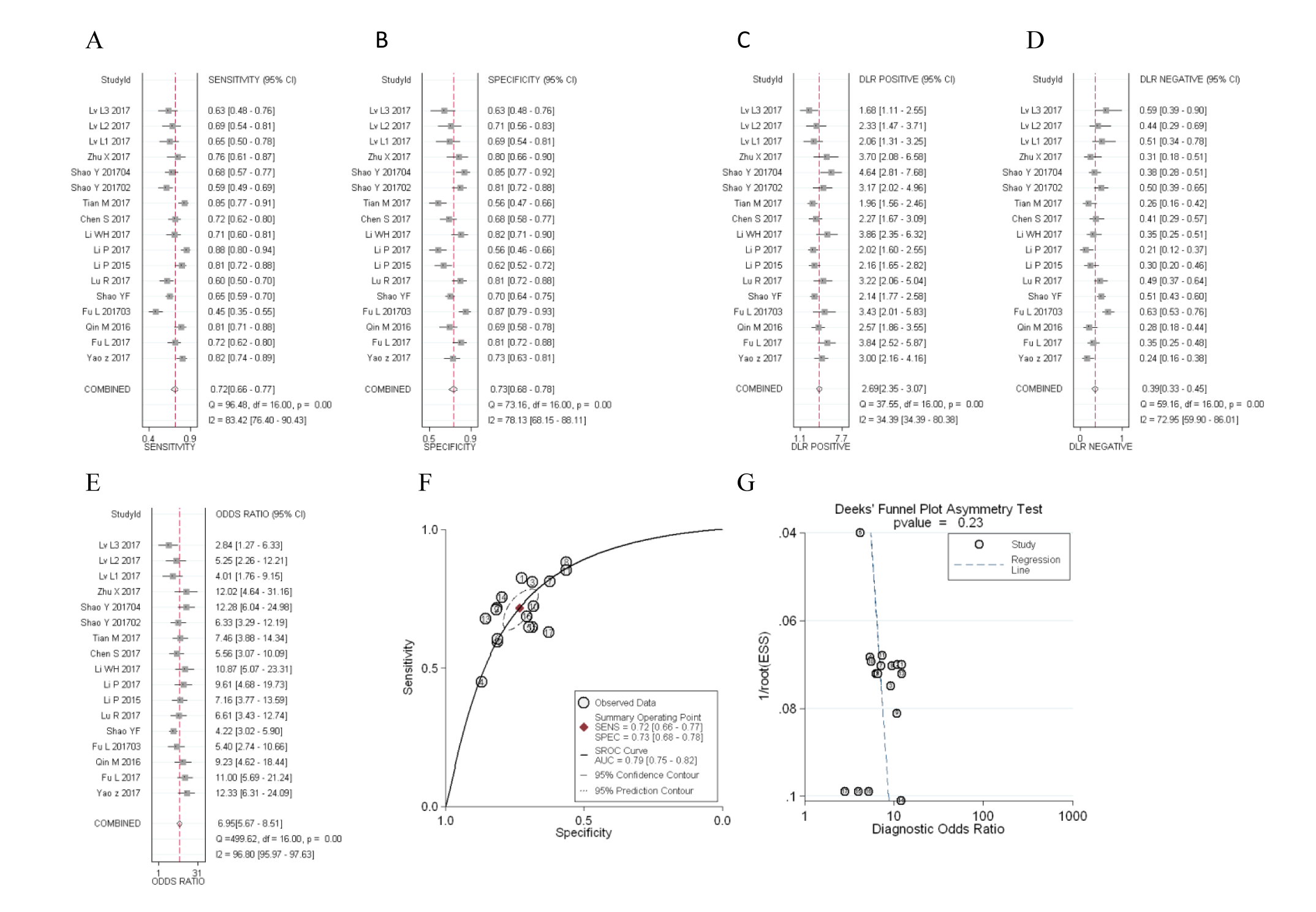

Supplement: Supplementary file 4 — Figure S3. Forest plots of PLR, NLR, DOR, AUC, and funnel plot for diagnosis of circRNA in tumors among 17 studies, which excluded 2 studies because of relatively small sample size (less than 50). (A) Sensitivity; (B) Specificity; (C) PLR; (D) NLR; (E) DOR; (F) AUC; and (G) Funnel plot. (TIFF 1858 kb) [file 12885_2018_4213_MOESM4_ESM.tif]
